# Author Correction: Continent-wide tree fecundity driven by indirect climate effects

**DOI:** 10.1038/s41467-021-22025-2

**Published:** 2021-03-08

**Authors:** James S. Clark, Robert Andrus, Melaine Aubry-Kientz, Yves Bergeron, Michal Bogdziewicz, Don C. Bragg, Dale Brockway, Natalie L. Cleavitt, Susan Cohen, Benoit Courbaud, Robert Daley, Adrian J. Das, Michael Dietze, Timothy J. Fahey, Istem Fer, Jerry F. Franklin, Catherine A. Gehring, Gregory S. Gilbert, Cathryn H. Greenberg, Qinfeng Guo, Janneke HilleRisLambers, Ines Ibanez, Jill Johnstone, Christopher L. Kilner, Johannes Knops, Walter D. Koenig, Georges Kunstler, Jalene M. LaMontagne, Kristin L. Legg, Jordan Luongo, James A. Lutz, Diana Macias, Eliot J. B. McIntire, Yassine Messaoud, Christopher M. Moore, Emily Moran, Jonathan A. Myers, Orrin B. Myers, Chase Nunez, Robert Parmenter, Sam Pearse, Scott Pearson, Renata Poulton-Kamakura, Ethan Ready, Miranda D. Redmond, Chantal D. Reid, Kyle C. Rodman, C. Lane Scher, William H. Schlesinger, Amanda M. Schwantes, Erin Shanahan, Shubhi Sharma, Michael A. Steele, Nathan L. Stephenson, Samantha Sutton, Jennifer J. Swenson, Margaret Swift, Thomas T. Veblen, Amy V. Whipple, Thomas G. Whitham, Andreas P. Wion, Kai Zhu, Roman Zlotin

**Affiliations:** 1 Nicholas School of the Environment, Duke University, Durham, NC USA; 2 INRAE, LESSEM, University Grenoble Alpes, Saint-Martin-d’Heres, France; 3 Department of Geography, University of Colorado Boulder, Boulder, CO USA; 4 School of Natural Sciences, University of California, Merced, Merced, CA USA; 5 Forest Research Institute, University of Quebec in Abitibi-Temiscamingue, Rouyn-Noranda, QC Canada; 6 Department of Systematic Zoology, Faculty of Biology, Adam Mickiewicz University, Poznan, Poland; 7 USDA Forest Service, Southern Research Station, Monticello, AR USA; 8 USDA Forest Service Southern Research Station, Auburn, AL USA; 9 Natural Resources, Cornell University, Ithaca, NY USA; 10 Institute for the Environment, University of North Carolina at Chapel Hill, Chapel Hill, NC USA; 11 Greater Yellowstone Network, National Park Service, Bozeman, MT USA; 12 USGS Western Ecological Research Center, Three Rivers, CA USA; 13 Earth and Environment, Boston University, Boston, MA USA; 14 Finnish Meteorological Institute, Helsinki, Finland; 15 Forest Resources, University of Washington, Seattle, WA USA; 16 Department of Biological Science, Northern Arizona University, Flagstaff, AZ USA; 17 University of California, Santa Cruz, Santa Cruz, CA USA; 18 USDA Forest Service, Bent Creek Experimental Forest, Asheville, NC USA; 19 USDA Forest Service Southern Research Station, Eastern Forest Environmental Threat Assessment Center, Research Triangle Park, NC USA; 20 Department of Biology, University of Washington, Seattle, WA USA; 21 School for Environment and Sustainability, University of Michigan, Ann Arbor, MI USA; 22 Department of Biology, University of Saskatchewan, Saskatoon, SK Canada; 23 Health and Environmental Sciences Department, Xian Jiaotong-Liverpool University, Suzhou, China; 24 Hastings Reservation, University of California Berkeley, Carmel Valley, CA USA; 25 Department of Biological Sciences, DePaul University, Chicago, IL USA; 26 Department of Wildland Resources, Utah State University Ecology Center, Logan, UT USA; 27 Department of Biology, University of New Mexico, Albuquerque, NM USA; 28 Pacific Forestry Centre, Victoria, BC Canada; 29 Université du Québec en Abitibi-Témiscamingue, Rouyn-Noranda, Quebec Canada; 30 Department of Biology, Colby College, Waterville, ME USA; 31 Department of Biology, Washington University in St. Louis, St. Louis, MO USA; 32 University of New Mexico, Albuquerque, NM USA; 33 Department for the Ecology of Animal Societies, Max Planck Institute of Animal Behavior, Konstanz, Germany; 34 Valles Caldera National Preserve, National Park Service, Jemez Springs, NM USA; 35 Fort Collins Science Center, Fort Collins, CO USA; 36 Department of Natural Sciences, Mars Hill University, Mars Hill, NC USA; 37 Department of Forest and Rangeland Stewardship, Colorado State University, Fort Collins, CO USA; 38 Ecology and Evolutionary Biology, University of Toronto, Toronto, ON Canada; 39 Department of Biology, Wilkes University, Wilkes-Barre, PA USA; 40 Geography Department and Russian and East European Institute, Bloomington, IN USA

**Keywords:** Ecology, Ecology

Correction to: *Nature Communications* 10.1038/s41467-020-20836-3, published online 23 February 2021.

The original version of this Article contained an error in Equation (1). The equation incorrectly included a “+” sign within the “growth-climate interactions” term. This has now been corrected in both the PDF and HTML versions of the Article.

