# Supplementary material for: Continent-wide tree fecundity driven by indirect climate effects
Source: Nat Commun. 2021 Feb 23;12:1242. doi: 10.1038/s41467-020-20836-3 (PMC7902660; doi:10.1038/s41467-020-20836-3)
Supplement: Supplementary file 1 — Description of Additional Supplementary Files [file 41467_2020_20836_MOESM1_ESM.pdf]

Description of Additional Supplementary Files

File Name: Supplementary Data 1

Description: Information for sites shown in Figure 1 of the main text listed by ecoRegion. Data type indicates crop counts (CC) or seed traps (ST). The PI(s) column lists coauthors of this study.

File Name: Supplementary Data 2

Description: Sample sizes by species in inventory data and MASTIF network, listed by family. Variables are defined in Table 1 of the main text. Sample-sizes are I trees: trees on inventory plots; M trees: number of trees on MASTIF plots; M plots: number of MASTIF plots; M tree-yr: number of MASTIF treeyears. Symbols highlight species-predictor combinations having zero below or above the 95% credible interval ('++' and '---', respectively) or the 68% credible interval ('+' and '-', respectively)

Gaaiho PDF Trial
www.gaaiho.com
