# Supplementary material for: Continent-wide tree fecundity driven by indirect climate effects
Source: Nat Commun. 2021 Feb 23;12:1242. doi: 10.1038/s41467-020-20836-3 (PMC7902660; doi:10.1038/s41467-020-20836-3)
Supplement: Supplementary file 4 — Reporting Summary [file 41467_2020_20836_MOESM4_ESM.pdf]

nature research

Corresponding author(s): *Double-blind peer review submissions: write DBPR and your manuscript number here instead of author names.*

Last updated by author(s): *YYYY-MM-DD*

Reporting Summary

Nature Research wishes to improve the reproducibility of the work that we publish. This form provides structure for consistency and transparency in reporting. For further information on Nature Research policies, see our Editorial Policies and the Editorial Policy Checklist.

Statistics

For all statistical analyses, confirm that the following items are present in the figure legend, table legend, main text, or Methods section.

n/a	Confirmed	
☐	☒	The exact sample size (n) for each experimental group/condition, given as a discrete number and unit of measurement
☐	☒	A statement on whether measurements were taken from distinct samples or whether the same sample was measured repeatedly
☐	☒	The statistical test(s) used AND whether they are one- or two-sided<br>*Only common tests should be described solely by name; describe more complex techniques in the Methods section.*
☐	☒	A description of all covariates tested
☐	☒	A description of any assumptions or corrections, such as tests of normality and adjustment for multiple comparisons
☐	☒	A full description of the statistical parameters including central tendency (e.g. means) or other basic estimates (e.g. regression coefficient) AND variation (e.g. standard deviation) or associated estimates of uncertainty (e.g. confidence intervals)
☒	☐	For null hypothesis testing, the test statistic (e.g. F, t, r) with confidence intervals, effect sizes, degrees of freedom and P value noted<br>*Give P values as exact values whenever suitable.*
☐	☒	For Bayesian analysis, information on the choice of priors and Markov chain Monte Carlo settings
☐	☒	For hierarchical and complex designs, identification of the appropriate level for tests and full reporting of outcomes
☒	☐	Estimates of effect sizes (e.g. Cohen's d, Pearson's r), indicating how they were calculated

Our web collection on statistics for biologists contains articles on many of the points above.

Software and code

Policy information about availability of computer code

Data collection	Data were obtained from published studies.
Data analysis	The code availability statement in the paper is here: Code in R and C++ is available on CRAN at https://cran.r-project.org/web/packages/mastif/index.html as mastif version 1.0.1, with additional background here: https://rpubs.com/jimclark/281413.

For manuscripts utilizing custom algorithms or software that are central to the research but not yet described in published literature, software must be made available to editors and reviewers. We strongly encourage code deposition in a community repository (e.g. GitHub). See the Nature Research guidelines for submitting code & software for further information.

Data

Policy information about availability of data

All manuscripts must include a data availability statement. This statement should provide the following information, where applicable:

- Accession codes, unique identifiers, or web links for publicly available datasets
- A list of figures that have associated raw data
- A description of any restrictions on data availability

Data from the study are available at https://doi.org/10.7924/r4348ph5t at the Duke Research Data Repository.

Field-specific reporting

Please select the one below that is the best fit for your research. If you are not sure, read the appropriate sections before making your selection.

☐ Life sciences ☐ Behavioural & social sciences ☒ Ecological, evolutionary & environmental sciences

For a reference copy of the document with all sections, see nature.com/documents/nr-reporting-summary-flat.pdf

Ecological, evolutionary & environmental sciences study design

All studies must disclose on these points even when the disclosure is negative.

Study description	As provided in the Supplement, the study contains observations from multiple sites, which included 211,146 trees and 2,566,594 tree-years from 123 species. Data were gathered from all suitable published and unpublished data available to authors. Locations are detailed in Table S3.2. Sample sizes, citations, data types are in Table S3.3.
Research sample	A "sample" in this study is a "tree-year". Dependence between tree years (between trees and within trees over time) is taken up by latent states in the model, detailed in the paper cited as Clark et al. 2019 (Ecol Monogr).
Sampling strategy	As is typical for Bayesian analysis, it is important to include all data. The dependence between different types of observations (seed traps, crop counts) is accommodated by conditional independence, i.e., a stochastic treatment of underlying states.
Data collection	Seed trap data are collected from traps emptied several times per year. Crop count data are obtained with binoculars.
Timing and spatial scale	All data meeting our requirements were included, starting in 1960 to the present. Observations are annual, assigned to an individual crop year.
Data exclusions	Data could not be included that are not aligned with an individual tree. For example, seed estimates for a stand could not be used (Clark et al. 2019).
Reproducibility	Clark et al. 2019 details data simulation, parameter recovery, and data prediction. The MASTIF package on cran was designed to allow the user to carry out these experiments herself.
Randomization	Groups in the study included individual trees (random--they are taken as 'exchangeable') and years (fixed effects, but random across ecoregions). Details are provided in Clark et al. (2019).
Blinding	NA

Did the study involve field work? ☒ Yes ☐ No

Field work, collection and transport

Field conditions	Crop counts require adequate visibility.
Location	These are detailed in Table S3.2.
Access & import/export	This was done by individual PIs.
Disturbance	Seed traps constitute infrastructure, but are viewed as non-invasive.

Reporting for specific materials, systems and methods

We require information from authors about some types of materials, experimental systems and methods used in many studies. Here, indicate whether each material, system or method listed is relevant to your study. If you are not sure if a list item applies to your research, read the appropriate section before selecting a response.

Materials & experimental systems

n/a	Involved in the study	
☒	☐	Antibodies
☒	☐	Eukaryotic cell lines
☒	☐	Palaeontology and archaeology
☒	☐	Animals and other organisms
☒	☐	Human research participants
☒	☐	Clinical data
☒	☐	Dual use research of concern

Methods

n/a	Involved in the study	
☒	☐	ChIP-seq
☒	☐	Flow cytometry
☒	☐	MRI-based neuroimaging
